# Evaluation of the feasibility, acceptability, and impact of Group Antenatal Care at the health post level on continuation in antenatal care and facility based delivery in Ethiopia using a cluster randomized stepped-wedge design: Study protocol

**DOI:** 10.12688/gatesopenres.15190.2

**Published:** 2024-10-14

**Authors:** Walelegn W. Yallew, Rediet Fasil, Della Berhanu, Konjit Wolde, Dedefo Teshite, Reena Sethi, Gayane Yenokyan, Yenealem Woldemariam, Stephanie Suhowatsky, Anne Hyre, Lisa Noguchi, Alemayehu Worku

**Affiliations:** 1 Addis Continental Institute of Public Health, Addis Ababa, Ethiopia; 2 Jhpiego Ethiopia, Assia Ababa, Ethiopia; 3 Jhpiego, Baltimore, MD, 21231, USA; 4 Johns Hopkins Bloomberg School of Public Health, Baltimore, MD, 21205, USA

**Keywords:** Health post, antenatal care, group antenatal care, health extension worker, Ethiopia, maternal outcomes, rural, facility-based delivery

## Abstract

**Background:**

Adequate antenatal care (ANC) and facility-based delivery are linked to improved maternal and neonatal outcomes. Adequate antenatal care attendance and facility birth rates are increasing in Ethiopia but remain well below national goals and global recommendations. Group ANC (G-ANC), when implemented at higher level facilities, is associated with improved quality and experience of ANC, and increased ANC retention and facility-based delivery. The objectives of the study are to assess the feasibility, acceptability, and impact of G-ANC implemented at lower-level facilities (health posts) on ANC continuation and facility-based delivery.

**Methods:**

G-ANC will first be piloted in five purposively selected health posts. The study will then use a stepped-wedge design in 36 health posts under six health centers, with randomization of the order of the start of the intervention done at the health center level (clusters). The design will include three time periods: first is a six-month control period with no G-ANC implementation, followed by another six months period where G-ANC will be introduced in half (n=18) of the study health posts, then final six months where G-ANC will be implemented in the remaining 18 health posts. Quantitative and qualitative data collection approaches will be used. The study has “pause and reflect” points designed to iterate on the intervention before rolling out to the next set of sites. Qualitative research will be conducted using in-depth interviews with pregnant women, health care workers, facility managers, and regional health managers. 770 women will be enrolled across all phases.

**Conclusions:**

The study will inform decision makers locally and globally on whether G-ANC is a feasible service delivery model at the health post level. Effectiveness of G-ANC at increasing ANC retention and facility-based delivery will be reported, as well as its acceptability to pregnant women and Health Extension Workers. Registration NCT05054491, ClinicalTrials.gov (September 23rd 2021).

## Introduction

Antenatal care (ANC) uptake is a key indicator to monitor progress towards improving maternal outcomes ( World Health Organization, 2016a). Although improvements have been made in the uptake of at least one ANC contact in Ethiopia, attendance for the recommended number of contacts remains low. The 2016 Ethiopia Demographic and Health Survey (EDHS) found 62% of pregnant women aged 15–49 received any ANC from a skilled provider, 32% of women had at least four ANC contacts, and 26% gave birth in a facility ( CSA/Ethiopia & ICF, 2017).

In Ethiopia, women are expected to receive ANC from both health centers (HCs) and health posts (HPs) during a pregnancy ( Assefa *et al.*, 2019). HCs provide comprehensive maternal health services, and HPs deliver primary health care packages, including ANC ( Gebretsadik *et al.*, 2019). Each health center typically has up to five affiliated HPs in its catchment area. Health posts are staffed by Health Extension Workers (HEWs) who are salaried female community health workers recruited from the community ( Kea *et al.*, 2018; Tsegay *et al.*, 2013). HEWs complete a one-year basic health service delivery course, and about 50% have received an additional one year of training ( Ministry of Health Ethiopia, 2022). HEWs are expected to spend 75% of their time in communities. At least two HEWs are assigned to one HP to serve a population of 3,000 to 5,000 persons in a village (kebele). Since 2020, the Ministry of Health (MOH) has taken steps to move more ANC services, including diagnostics, to the health post level ( FMoH, 2020). Each health post (where HEWs work) is generally supported by health workers from the nearby health center, who provide supervision, technical support, and, when necessary, clinical services that are beyond the scope of HEWs. Typically, HEWs refer cases that need more advanced care at health centers. The MOH supports several community-based interventions to increase awareness about danger signs and the importance of skilled care at birth, including mobilization of unpaid female volunteers (i.e., Women’s Development Army leaders). Also, pregnant women conferences are coordinated by HEWs and conducted monthly at the village level by a nurse/midwife ( Asresie & Dagnew, 2019).

The World Health Organization (WHO) recommends group antenatal care (G-ANC) in the context of rigorous research as an alternative to individual ANC ( World Health Organization, 2016b). G-ANC is a transformative service delivery model that provides care to groups of 8–12 pregnant women of similar gestational age through a series of scheduled meetings ( Grenier *et al.*, 2019). G-ANC incorporates physical assessment, education and skill development, and peer support and takes a broader, more holistic, woman-centered approach to traditional antenatal care ( Sharma *et al.*, 2018). Several studies have found that G-ANC is associated with increased ANC attendance; improved health literacy and client satisfaction; increased uptake of family planning; improved birthweight; and increased breastfeeding initiation/duration ( Hale *et al.*, 2014; Manant & Dodgson, 2011; McKinnon *et al.*, 2020).

This study will evaluate the acceptability, feasibility, and effectiveness of group ANC (G-ANC) at the health post level compared to usual ANC among women who report intention to receive ANC at the health post level. It builds on existing local efforts and addresses important questions regarding how ANC service delivery might be modified to improve quality (both provision and experience) of care. This proposed study will introduce G-ANC at a lower level of the health system (i.e., the health post) to address a key element of the global G-ANC learning agenda: can the model feasibly be delivered by community-based providers closer to women’s own homes and communities, while retaining quality of care and other positive outcomes associated with G-ANC at higher level facilities ( Grenier *et al.*, 2020). The proposed G-ANC intervention at the HP level will be led by HEWs. G-ANC will build from group education activities at health posts and support service delivery reorganization into G-ANC to test potential improvements in ANC attendance and facility-based delivery, both of which are MOH priorities.

## Methods

### Study design

**Figure 1.**
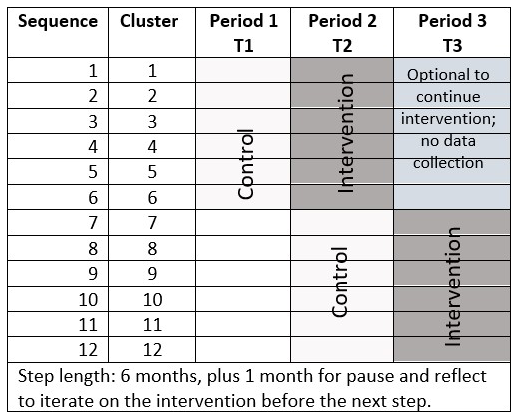
Time Steps and Clusters.

The mixed-methods study will use a stepped wedge cluster randomized controlled trial with randomization of the order of the start of the intervention done at the health center level (clusters) in the Amhara region. The study will begin with a pilot to test the intervention conducted by HEWs at the health post level. If feasible, randomized controlled trial will be conducted with three time periods: baseline period (T1); time period 2 (T2); and time period 3 (T3), each about 6 months in duration. Control period for the first half of clusters will occur at T1. Half of the clusters will start G-ANC first (at T2), and the control period for the second half of the clusters will also occur at T2. The second half of the clusters will start the intervention in the third time period (T3). The first half of clusters will have a choice to continue G-ANC with a second cohort of women during T3 ( Figure 1). We plan to use a stepped wedge study design, because it allows for iterative learning (i.e., adaptive management) during the study to modify the intervention, which was novel compared to traditional cluster RCT. The study was registered on September 23, 2021 on clinicaltrials.gov with registration ID: NCT05054491.

Prior to the intervention, all women in the study HPs will receive the current standard of individual ANC. At the time of the intervention, the health posts in the selected clusters will provide G-ANC to one cohort of 8–10 pregnant women. G-ANC participants will be followed prospectively from the time of study enrollment. All non-enrolled pregnant women in the intervention health posts will continue to receive individual ANC.

### Study setting

The G-ANC study will be conducted in two zones of the Amhara regional state: West Gojjam Zone (South and North Achefer districts) and the South Gondar Zone (Farata, Dera, and Libokemem districts). Twelve health centers in these districts will be selected as G-ANC study centers. G-ANC meetings will take place at the health post level only. Amhara region has an estimated population of 22.5 million ( CSA/Ethiopia & ICF, 2017). Amhara region has 12 administrative zones, and the economy is primarily subsistence farming.

### Participants

Twelve HCs will be selected in the Amhara region after meeting the eligibility criteria ( Table 1) and categorized as follows: 1) type of location (peri-urban or rural); and 2) client volume for the last one-year (low or high) volume. Three of the five health posts per HC (total 36 health posts) will be selected to participate based on inclusion criteria. Study participants will be: pregnant women attending ANC; HEWs; and facility and regional managers working in study sites in Amhara region.

**Table 1.** Facilities and study participants inclusion and exclusion criteria.

Facilities and study participants inclusion and exclusion criteria		
**Health facilities**	Inclusion criteria	For health centers: • Located in Amhara, Ethiopia • Have health posts in the catchment area that provide point of care testing • Affiliated with functioning health posts where HEWs provide ANC • Permission granted by health facility management to participate in the study For health posts: • A minimum of two HEWs per health post (one of whom is a level four HEW with the additional year of training) • On-site availability of ANC services, including on-site point of care tests • Have adequate space to hold G-ANC meetings • Permission granted by health post management to participate in the study • Enroll a minimum of 10 new ANC clients per month who are <=20 weeks gestational age, as determined by the health management information system (HMIS) *(Subject to change based on the results of the pilot phase)*
**Health extension** **workers**	Inclusion criteria	• Working in a participating/selected health facility and providing ANC services • Willing to participate in the study • Based in the selected health post • Agree to provide G-ANC
**Health facility** **managers & Regional** **health managers**	Inclusion criteria	• Working in Amhara region for a period of at least 6 months prior to beginning of the study
**Pregnant women** **(ANC Clients)**	Inclusion criteria	• Minimum age of 15 years at the time of enrollment; pregnant 15–17-year-olds will be treated as emancipated/mature as per local regulations • Gestational age less than or equal to 20 weeks at the time of enrollment determined by last menstrual period (LMP), pelvic exam, fundal height, quickening, ultrasound, and/or timing of fetal heart tones and pregnancy test * • Pregnant women able and willing to provide adequate locator information • Planning to reside at their current location for at least 10 months • Agree to participate in the study and continue ANC at health post • Willing to participate and consent to follow up for up to 6 weeks post-delivery • Are willing to receive G-ANC at the health post level (during the intervention period)
	Exclusion criteria	• Women who plan to travel away from the study site for more than four consecutive weeks during ANC or after 6 weeks post-delivery • Women who are unable provide consent • Women who have any condition that in the opinion of the investigator or designee, would complicate interpretation of study outcome data, or otherwise interfere with achieving the study objectives

* Up to two additional pregnant women who meet study criteria can be added to a G-ANC cohort if added to the cohort by the second G-ANC meeting.

### Intervention

During the intervention time period, pregnant women who come for their first ANC visit at the health post will receive standard individual care. At the end of this first ANC visit, eligible women who agree to participate in G-ANC will be added to the G-ANC cohort. Approximately 8–10 women will be enrolled in a cohort. Up to two additional pregnant women who meet study criteria can be added to a G-ANC cohort if added to the cohort by the second G-ANC meeting. Six monthly G-ANC meeting will be conducted for each cohort on a fixed day/time at each Health Post(HP). Cohort membership will be fixed (i.e., the same women and facilitators at each meeting). Each cohort will meet for G-ANC meetings in a designated meeting space, set up to ensure privacy during clinical consultations. G-ANC meeting will be conducted by at least one HEW, who is trained in the G-ANC service delivery model.

Each meeting will have the same general structure: self-assessment by the clients themselves and initially taught and supervised by the HEW (such as measurement of weight and blood pressure using an automated cuff); discussion around pre-determined themes using materials designed for G-ANC; individual assessments by the HEW in a private space; and peer support activities that create group cohesion. Table 2 presents the G-ANC meeting schedule and content per meeting. Each meeting is expected to last 90–120 minutes and will be documented as an ANC contact. Social distancing in response to COVID-19 as per national guidelines will be enforced during meetings. Women will have the opportunity for additional private time with the HEW after the G-ANC meeting if desired or needed for health and/or safety. Women will be told that they can and should return at any time if they have questions or concerns. Phone/text reminders will be used to remind participants of G-ANC meetings as a strategy to improve ANC attendance.

**Table 2.** G-ANC intervention meeting schedule.

MEETINGS	TIMING (GA)	THEME/MEETING PLAN
Meeting 1	4–5 months	• Introduction to group care • Preventing problems in pregnancy • Teach self-assessments
Meeting 2	5–6 months	• Danger signs during pregnancy • Sexually transmitted infection (STI) transmission and partner communication/negotiation
Meeting 3 partners encouraged to attend	6–7 months	• STI transmission & partner testing • Birth plan, complication readiness • Healthy timing and spacing of pregnancy
Meeting 4	7–8 months	• Danger signs during labor and preventing problems after birth
Meeting 5	8–9 months	• Danger signs after birth
Meeting 6	9+ months	• Newborn care and danger signs

The G-ANC intervention will be implemented at the health posts that offer integrated point-of-care testing (POCT) to screen for anemia, syphilis, and asymptomatic bacteriuria (ASB). Availability of these rapid test kits improve the quality of ANC and reduce the need for women to travel to the health center for routine screening. Point-of-care testing was introduced and piloted in these facilities, but it is not yet the national standard of care for ANC. The test kits were available in all HPs at the start of the study and will be provided by the study until completion.

The G-ANC intervention and materials were adapted from Jhpiego’s G-ANC intervention model, which consisted of five monthly two-hour meetings with clinical assessments alongside structured group discussions and activities and facilitated by nurses, midwives, or community health extension workers ( Grenier *et al.*, 2019). It was modified in collaboration with the MOH for the Ethiopian context as a six-meeting model. The model is designed to increase early entry into ANC and increase retention in ANC, in support of changes to the Ethiopian national ANC guidelines that increased from 4 to 8 ANC contacts ( Ministry of Health Ethiopia, 2022). Intervention materials in Amharic language include: a pictorial self-assessment card women used at the beginning of each meeting to record the weight, blood pressure and indicate any danger signs; meeting guide used by the facilitators to guide each meeting; 5–6 A4-size illustrated picture cards per meeting that are designed for nonliterate audiences (total 36 cards), with questions on the back of each card to foster discussion; and a A4 booklet with small versions of all the picture cards that women can take home to share what they’ve learned with their spouses and families; an implementation guide for HEWs to introduce G-ANC in their health post; a simple G-ANC register is part of the intervention package to aid the facilitator to track attendance and record other data; and three monitoring tools, including a meeting observation checklist to provide feedback and assess fidelity. The picture cards were pre-tested with HEWs before the pilot phase, and all the intervention materials will be revised as needed based on the pilot feedback. Each intervention HP will be provided these materials, 12 chairs, a basic set of supplies to run activities, two weigh scales, three user-friendly Microlife PSA blood pressure devices for the self-assessments, and one privacy screen to divide the private clinical consultation area from the larger G-ANC meeting space.

All clients will continue to receive the components of standard care, and no standard therapies will be denied to the intervention group. Likewise, standard, individual ANC care will not be denied if requested at any time.

***Training:*** At the start of each time period, two HEWs in each intervention HP will be trained on the G-ANC intervention (five days) in a highly participatory, facilitated learning environment by study staff. The HEWs will also be trained (1.5 days) on estimating gestational age including hands-on practice to improve identification of eligible women. Midwives from HCs who will provide onsite mentorship to HEWs during G-ANC implementation will be trained for four days on G-ANC focused mentorship by the study team. Key stakeholders, including participating health facility managers and regional managers, will be oriented to G-ANC to build ownership and support.

### Outcomes

***Primary outcome 1*:** Proportion of women with at least four ANC visits.

***Primary outcome 2*:** Proportion of women who delivered at health facility.

After exploring the balance by time period/intervention status and correlations, the effect of G-ANC will be estimated using generalized linear mixed effects models with binomial distribution and logit-link.

We will adjust for facility- and individual-level characteristics that show imbalance between the intervention vs. control time periods and can be strongly correlated with the outcome scores. The primary analysis will not adjust for multiplicity and will be performed at 0.05 level of statistical significance.

***Secondary outcomes*:** Satisfaction level of women with ANC service, proportion of women who reported self-efficacy or empowerment.

Analysis of secondary outcome measures will follow a similar plan as the analysis of the primary outcome. Subgroup analysis will be conducted for specific subgroups to determine if the interventions were different. The key subgroups of interest are marital status, education, and economic status.

### Recruitment and consent

ANC clients will be recruited via four strategies: 1) pregnant women will be identified as potential study participants through existing local community structures (such as the women’s development army leaders) as a means to explain the study to potential participants and recruit them into the study; 2) All new ANC attendees in the intervention HPs will be screened for eligibility during the first ANC contact with a ANC service provider; 3) All new ANC attendees in the study HC will be screened for eligibility during the first ANC contact with a ANC service provider; 4) eligible women will be identified by G-ANC trained HEWs who make door-to-door visits in the community.

A research assistant will rotate among each of the intervention HPs during the recruitment period to explain the study to women who meet the inclusion criteria and agree to participate in the study.

The research assistants will obtain verbal consent from women in a private space in the clinic using IRB-approved recruitment and consent documents in the local language. Verbal consent will be sought instead of written consent as written consent is seen as a personal identified in the community and verbal consent is preferred. This consenting method was approved by the ethics committee (see section Ethics). The potential G-ANC participants will be informed during the consenting process of the expected number of meetings (i.e., ANC visits), their rights as participants, risks, and benefits. The research assistant will explain the need to collect their names, phone numbers, and addresses, which will be used for study-related follow-up purposes only. Anyone declining participation in the study will be referred to individual ANC care during the intervention period. A baseline questionnaire will be administered to consenting study participants at the beginning of each step (time period).

For HEWs and health facility managers, study staff will explain the purpose based on the inclusion criteria. The HEWs will be oriented to the study including the randomization (they will not be blinded to the order of randomization of their health center and affiliated health posts) and their role in the study including screening ANC first time clients for eligibility to participate. HEWs who will be facilitating G-ANC meetings will be informed that they may be asked to participate in an in-depth interview (IDI) at the end of the study related to implementation of group-based care and its effect on their job and satisfaction levels. Oral consent will be obtained in a private location at the health facility by research assistants.

For the regional health managers, study staff will contact the Regional Health Bureau responsible for overseeing reproductive health programs in Amhara region via phone or in-person and invite the regional health manager to participate in an IDI as per the inclusion criteria. An appointment will be scheduled to obtain informed consent and conduct the IDI, preferably at the Regional Health Bureau’s office. Written consent will be obtained by the research assistant, and the interview conducted in a private location.

For the in-depth interviews of women and HEWs involved in G-ANC, qualitative data will be collected at the end of the study after all G-ANC meetings have been conducted. Oral consent will be obtained from the HEWs and eligible women 3–6 months postpartum to be enrolled for the qualitative portion of the study.

### Harms

The intervention in this study is for women to take part in group ANC. Some of the components of the intervention is focused on nutrition, birth preparedness, breastfeeding, and postpartum care Some risks, discomforts, and inconveniences may apply and will be minimized as outlined in Table 3.

**Table 3.** Risks, discomforts, and inconveniences.

Risk/discomfort/inconvenience	Response to Minimize/Mitigate
**Difference in quality of care** **compared to traditional one on one** **ANC**	Although it is expected that this intervention will improve quality of care, there is a small risk that women in G-ANC could experience lower quality of care related to routine/conventional ANC with HEWs. This will be mitigated by encouraging all women to return at any other non-group time if they have questions or concerns.
**Discomfort/emotional distress** **from discussing sensitive or** **difficult topics**	Potential topics of discussion during group time include experience with poor maternal or neonatal outcomes; gender-based violence; prevention/treatment of HIV; and family planning. Women will never be directly asked of their own status or personal experience. These topics will be discussed in terms of what participants have seen or heard so that discussion can occur in the abstract and need not directly reflect the women present. In addition, women will be encouraged to share only what they are comfortable sharing and facilitators will be trained to respond to difficult emotional situations.
**Time spent at the health post**	It is possible that women who attend G-ANC may be inconvenienced due to the longer time spent at the health post to participate in the meetings.
**Breach of confidentiality by other** **members of group**	If women do opt to share personal information it is possible that that information will be shared by other group members. To mitigate this, group norms will be discussed at every meeting to reinforce that the group meetings are confidential, and information about other women should not be shared outside the group without the woman’s explicit consent. Individual test results will never be shared in the group setting, but rather during the brief private assessment done during each meeting. This is also a time where women can discuss any issues, they would like to remain confidential between themselves and the provider. However, confidentiality breaches have not been identified as a significant concern with G-ANC in the literature.
**Breach of confidentiality by study** **staff**	We will conduct comprehensive training in confidentiality and appropriate research/clinical standards for study staff before the study begins and reinforce this training throughout the study. We do not anticipate any breach of confidentiality on the part of the study staff. Any breach of confidentiality will be met with counseling and support provided by the study and clinical staff. In addition, any study staff responsible for a breach of confidentiality will be disciplined appropriately, up to and including termination of employment.
**Referral**	If a participant needs clinical referral as part of her care, this will be provided to both the control and intervention arms according to standard site protocols and will not be affected by the study.

### Sample size

We used the existing data from Amhara region for two primary study outcomes (i.e., four or more ANC contacts (31.5%) and facility-based delivery (27.1%) to calculate the number of clusters (i.e., HCs) to be randomized to be able to detect an odds ratio of 2 comparing post-intervention to pre-intervention proportion with 80% statistical power using stepped wedge design with three time periods and two steps for switching from standard care to intervention ( Figure 1). For the complete stepped-wedge design, the calculations indicate that a sample of 12 HCs with three time periods/two steps, six HCs switching from control to treatment at each step, and with an average of 30 women per cluster (i.e., three HPs with 10 women in each) per time period achieves 84% power to detect an odds ratio of 2 at 0.05 level of statistical significance. The underlying pre-intervention proportions and the post-intervention proportions associated with odds ratio of 2 are shown in Table 4.

**Table 4.** Pre-intervention and post-intervention proportions with associated odds ratio.

Study Outcome	Pre-Intervention Proportion, P2 ( Table 1)	Odds Ratio to be Detected	Post-Intervention Proportion, P1
Four or more ANC contacts	0.315 (Amhara)	2	0.48
Facility-based delivery	0.271 (Amhara)	2	0.43

The within-HC correlation (ICC) is assumed to be 0.100. This level of correlation is consistent with the one reported by Ousman *et al.*, where clustering was done at enumeration area level with an ICC of 0.11 for ANC visits ( Ousman *et al.*, 2019). The total sample size for ANC clients is then 12 HCs x 3 HPs x 10 women/HP x two time periods=720 women. An odds ratio of 2.0 translates to 50–60% improvement in completion of four or more ANC contacts and 60–70% improvement in facility-based delivery in the G-ANC arm compared to pre-intervention.

The study will be conducted in 12 health centers. The overall sample size is a total of 770 women. The pilot will enroll 50 women (i.e., 10 women per HP, 5 HPs), and the RCT will include a maximum of 720 women ( Table 5). Sample size for the qualitative data collection is indicated in Table 6.

**Table 5.** Number of Health center, Health post and women included in the study, in G-ANC arc project, Ethiopia.

	Pilot	Time parried 1 Control	Time Period 2 (control & Intervention)	Time Period 3 (Intervention)	Total
Health Center	2	6	12	6	**14**
Health Post	5	18	36	18	**41**
Women	50	180	360	180	**770**

**Table 6.** Summary of sample size for qualitative data collection.

Study population	Estimated number of in-depth interviews (IDIs) and key informant interviews (KIIs) per round of data collection (after pilot, after T2 and after T3)	Total
Women who completed most G-ANC meetings	Up to 9, in 3 rounds	27
Women who dropped out of G-ANC after 1–2 meetings	Up to 9, in 3 rounds	27
Women who completed most G-ANC meetings, but did not gave birth in a health facility	Up to 9, in 3 rounds	27
Women who completed most G-ANC meetings and gave birth in a health facility	Up to 9, in 3 rounds	27
HEWs	Up to 5, in 3 rounds	15
Health facility managers	Up to 1 per facility, 18 HPs T2 and 18 HPs T3	36
District/Regional managers	Up to 1 per woreda manager Up to 1 per regional manager	15 3

### Randomization

Health centers with catchment health posts that provide POCT for ANC services will be listed and randomized to begin the intervention at T2 (i.e., the first randomized set of facilities) or T3 (i.e., the second randomized set of facilities). These facilities will be randomized 1:1 before T1 to two different intervention start times (i.e. two sequences), stratified by location and client volume, using randomly permuted blocks of size 2 and 4. We will use centralized randomization as the allocation concealment mechanism: A remote and independent statistician in the Johns Hopkins Bloomberg School of Public Health (JHSPH) will generate and implement the allocation sequence, and communicate it to the study sites after confirming the eligibility of the clusters. HEWs will identify pregnant women and provide the list of women to ACIPH. ACIPH field workers will get consent and enroll eligible women.

***Blinding*:** Study staff and those assessing outcomes will not be blinded to knowing the study time period to which facility is randomized. The participants will also not be blinded about the type of care they will be receiving (group ANC or routine/conventional care).

### Study implementation

G-ANC at the health post level is a new intervention, therefore the study will be rolled out using a phased approach.

***Pilot phase*:** Prior to study activation, we will pilot G-ANC in up to five purposively selected health posts in Amhara region that will not be included in the study. This pilot phase will allow us to adapt G-ANC materials to adjust for the literacy levels of the HEW and conditions at the health post. After the materials have been adapted, up to two HEWs per health post (total 10 HEWs) will be trained to facilitate G-ANC. In the pilot phase, we will enroll a maximum of 50 women (up to 10 per HP x up to 5 HPs). The pilot phase will also allow us to determine whether: HEWs are able to facilitate the G-ANC meetings; there are enough eligible ANC clients at the health post level; we are able to enroll enough women at the HP who are of a similar gestational age; and women come back to the HP for the G-ANC meetings. After completing at least two G-ANC meetings in the pilot, we will determine the feasibility of moving to Phase 2 of the study.

**Figure 2.**
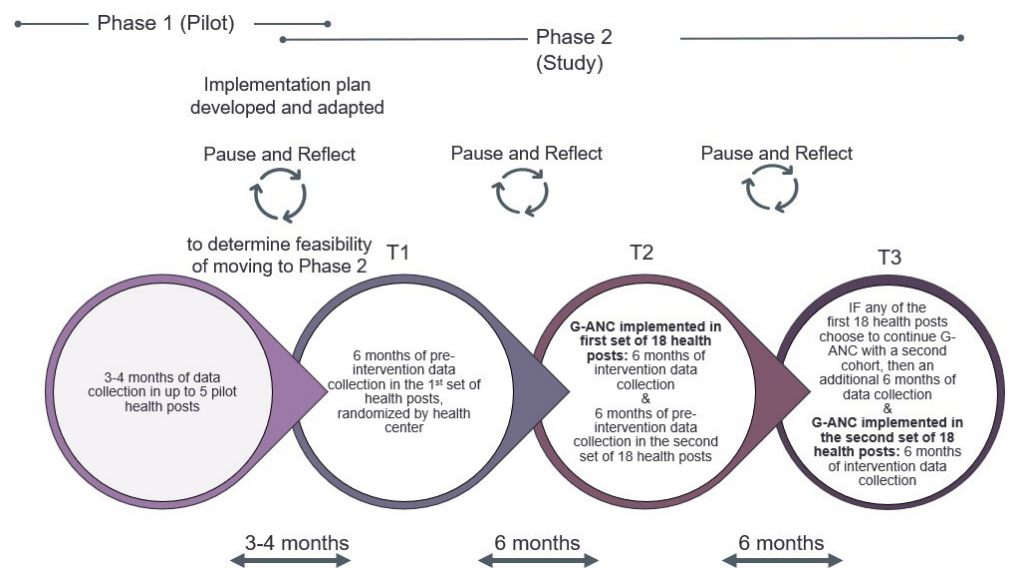
Study Timeline with pilot and trial phases.

***Trial phase*:** Phase 2 includes T1, T2, and T3. During T1, pre-intervention data will be collected from the first set of 18 HPs by interviewing pregnant women during their pregnancy and again postpartum. The women in T1 will not receive the intervention. During T2, the G-ANC intervention will be implemented in the first set of 18 HPs (first 6 clusters), and pre-intervention data collection will be conducted in the second set of 18 HPs (i.e., second 6 clusters). During T3, the G-ANC intervention will be implemented in the second set of 18 HPs. If the first set of 18 HPs chooses to continue the G-ANC intervention with a second cohort, an additional six months of data collection will occur during T3. Participant timeline is depicted on Figure 2.

Once study participants are enrolled, the participant timeline is about 8.5 months: enrollment with a baseline questionnaire; follow-up for about six months during ANC; and end-line survey up to 6 weeks post-delivery. Participants in the qualitative research will have a single contact for the IDIs.

The overall duration of the entire study is 18 months. The implementation process will be documented in these sites, and a transition plan will also be developed for Jhpiego to handover implementation to the government.

### Participant retention

Implementation of a paper-based participant tracking system will be used to accurately assessing participant retention in the study. The G-ANC registration and participating registration book will be used to track ANC attendance. This will provide the HEWs with a listing of all participants who have missed scheduled G-ANC meetings.

### Data collection

Both quantitative and qualitative data collection approaches will be used in this study. The following data will be collected in the pilot phase and then during study implementation (Phase 2).

Once enrollment begins, all new ANC attendees in the selected health posts will be screened for eligibility during the first ANC contact with a health care provider. Screening and Enrollment Questionnaire will be completed. Basic demographic data will be collected from all eligible consented women (all who meet the inclusion criteria). All data will be de-identified for those who decline study participation. Baseline and endline data will be collected from all eligible and voluntary women participating in all study time periods. During the first period, data will be collected from 18 control health posts. In the second period (T2), data will be collected from 18 intervention and 18 control health posts. In the third period, (T3) data will be collected from 18 intervention health posts.

Data quality will be monitored through the use of trained research assistants and supervisors, data quality supervision from, and use of an electronic data collection program to minimize data entry errors. The study team will select respondents for the IDIs at the end of the study based on preliminary findings from the quantitative survey. IDIs with women will be conducted at the end of T2 and T3, after completion of all of the G-ANC meetings ( Table 7). Specific topics/domains to explore with G-ANC participants include: satisfaction with care; any perceived changes in self-efficacy or empowerment; health literacy; improved communication and/or trust with providers and the health system as a whole; factors in uptake/non-uptake of postpartum family planning (PPFP); and the overall impression of the G-ANC model. In this study, we used standardized pretested tools to assess the levels of empowerment, self-efficacy, and satisfaction. For empowerment and self-efficacy, we will use 8 questions related to their current pregnancy, and then calculate a composite score to understand the mothers' level of empowerment. We asked 11 questions on satisfaction during the last antenatal care will be asked about the antenatal care received during your last pregnancy and the current pregnancy for this GANC

**Table 7.** Data collection timing, per tool.

Data collection tool	Study participant	Timing of data collection		
		T1	T2	T3*
Cluster level information	Health Post level Information	18 HPs	36 HPs	18 HPs
Tool 1a. Screening and enrollment questionnaire	Pregnant women (ANC clients),	18 HPs (180 women) Control	36 HPs (360 (intervention and control)	18 HPs (intervention) starting G-ANC
Tool 1b. Screening and enrollment log		18 HPs (180 women) Control	36 HPs (360 (intervention and control)	18 HPs (intervention) starting G-ANC
Tool 2. Baseline client survey at ANC1		18 HPs (180 women) Control	36 HPs (360 (intervention and control)	18 HPs (intervention) starting G-ANC
Tool 3. Endline client survey		18 HPs (180 women) Control	36 HPs (360 (intervention and control)	18 HPs (intervention) starting G-ANC
Tool 4. G-ANC enrollment register	Pregnant women (ANC clients), intervention only	NA	180 Women enrolled G-ANC	180 Women enrolled G-ANC
Tool 5. In-depth interview guide, ANC participants	Pregnant women (ANC clients), intervention and control HPs	NA	Time period two end line (intervention site)	Time period three end line (intervention site)
Tool 6. In-depth interview guide, HEWs	HEWs, intervention and control HPs	NA	Time period two end line (intervention site)	Time period three end line (intervention site)
Tool 7. In-depth interview guide, Health managers	District Managers, Health facility Managers intervention and control HPs	NA	Time period two end line (intervention site)	Time period three end line (intervention site)

IDIs will be conducted with HEWs who were trained in G-ANC and facilitated G-ANC meetings in the intervention sites and health managers. Specific topics/domains to explore with providers will include: attitudes towards providing group care; perceived changes in their communication and relationship with patients (if any); changes in empowerment, self-efficacy, and satisfaction related to their perceived ability to do their job well; changes in workloads; sustainability of group care; suggested changes to the model /logistics required to offer group care; perceived effects of group care on colleagues and patients. IDIs with health facility managers and regional health managers will be conducted at the end of T2 and T3 in the intervention areas.

### Data analysis

Data analysis and the reporting of results for this study will be conducted in accordance with norms for analyzing cluster randomized trials as described in the Consolidated Standards of Reporting Trials guidelines with considerations for the step-wedge extension ( Hemming *et al.*, 2018; Hemming *et al.*, 2019).

***Quantitative data analysis*.** The primary analysis will be conducted using intention-to-treat (ITT) analysis. After exploring balance by time period/intervention status and correlations, the effect of G-ANC will be estimated using generalized linear mixed effects models with binomial distribution and logit-link. The model will include G-ANC as the intervention indicator, time period and their interaction as the primary predictors, and HC as the random intercept. We will adjust for facility- and individual-level characteristics that show imbalance between the intervention vs. control time periods and can be strongly correlated with the outcome scores. The amount of missing data will be assessed for each variable and overall, for the sample. If more than 5% of data on covariates are missing, multiple imputation procedures will be used assuming data are missing at random ( Sterne *et al.*, 2009).

***Qualitative data analysis*.** Following each audio recording, either the moderator or an official transcriber familiar with local language and English will transcribe from the audio recording. Analysis will be ongoing. Coding of textual passages will be done using open code 4.03 Umeå: Umeå University, an open-source tool known for qualitative analysis.

### Data management

Following the computer system validation, the data manager will proceed to build, test, validate the master plan, and document testing of the study database using the SurveyCTO platform. SurveyCTO will automatically capture quantitative data and synchronize with the server. SurveyCTO will be programmed so that automated quality checks and data classification systems will be run. Data will be maintained in a way that preserves their accuracy, integrity, and legibility with user access restrictions and data retrievable by designated personnel only. A security system that prevents unauthorized access to the data will be maintained. The database management system will be password protected, with each member of the research team responsible for data management having their own password. After data collection, Addis Continental and Johns Hopkins Bloomberg School of Public Health (JHSPH) will de-identify the data. Quality control reports will be sent to the study team bi-weekly indicating missing, overdue data and outstanding queries during the baseline and end-line survey time periods. Throughout the fieldwork, field supervisors and the research team will observe the research assistants conducting interviews and carry out field editing. By checking the research assistants’ work regularly, the field supervisor can ensure that the quality of the data collection remains high throughout the data collection period. The de-identified dataset will be made available on ClinEpiDB or another open access site.

### Ethics

Research assistants will be trained in the study in a multi-day training workshop, covering the important points of the approved study protocol, including privacy and confidentiality. We will review and discuss all relevant content in the JHSPH Ethics Field Training Guide. We will review data collection procedures, which will ensure ethical conduct of research and data integrity. Ethical approval was obtained from the Addis Continental Institute of Public Health institutional ethical review board, Addis Ababa, Ethiopia (ACIPH/IRB/006/2021); and the JHSPH Institutional Review Board, Baltimore, Maryland, United States of America (IRB14448). ACIPH and JHSPH IRB amendments will be obtained for important protocol modifications, if any.

## Dissemination of study results

The study team plans to disseminate findings among national and sub-national stakeholders through in-country dissemination events and globally through peer-reviewed journal manuscripts and international conference presentations. About five publications, including conference abstracts and journal manuscripts, are planned. Study findings will be presented to national and sub-national health officials to determine if and how G-ANC at the health post level will be integrated into policy and included as a strategy for service delivery. The study will contribute to the body of knowledge that will inform decision makers locally and globally on whether G-ANC is a feasible service delivery model at the health post level that is more acceptable and effective than individual ANC.

## Study status

The study is active. Quantitative baseline and endline surveys of T1 and T2 have been conducted. Currently, prepararations are underway for T3, but implementation of this step may be delayed until security improves in the region.

## Conclusion

The study will contribute evidence regarding whether G-ANC is a feasible service delivery model that is more acceptable and effective than individual ANC at the health post level. It will inform local and global stakeholders regarding whether this model is feasible and of sufficient value to adopt as policy and implement more broadly. The findings of this study are expected to inform decision-making at different levels on whether to adopt the model as a matter of policy, and how G-ANC can be integrated into routine service delivery.

## Data Availability

No data are associated with this article. Figshare: SPIRIT checklist for ‘Evaluation of the feasibility, acceptability, and impact of Group Antenatal Care at the health post level on continuation in antenatal care and facility based delivery in Ethiopia using a cluster randomized stepped-wedge design: Study protocol’, *https://doi.org/10.6084/m9.figshare.25434388.v1*, ( Yallew *et al.*, 2024). Data are available under the terms of the Creative Commons Attribution 4.0 International license (CC-BY 4.0).
