# Supplementary material for: Evaluation of the feasibility, acceptability, and impact of Group Antenatal Care at the health post level on continuation in antenatal care and facility based delivery in Ethiopia using a cluster randomized stepped-wedge design: Study protocol
Source: Gates Open Res. 2025 Jan 31;8:29. Originally published 2024 Apr 2. [Version 3] doi: 10.12688/gatesopenres.15190.3 (PMC11813170; doi:10.12688/gatesopenres.15190.3)
Supplement: Peer review history

# Reviewer report: Reviewer response for version 3

**DOI:** 10.21956/gatesopenres.17706.r39424

**Published:** 2025-03-03

**Authors:** Angela M Bengtson

**Affiliations:** 1 Department of Epidemiology, Emory University, Atlanta, Georgia, USA

Overall, the revised manuscript is much improved. A few comments related to data collection and analysis have not been adequately addressed:

- some discussion around how the pause and reflect sessions will be used to adjust intervention content and if and how that will be handled at the analysis phase is needed. If comparisons are only planned by intervention and control groups within a given time period (which needs clarification) then this may not be an issue. But if there is interest in analyzing all intervention period together and there are major changes to the intervention during the pause and reflect period, this would not be appropriate.
- facility and individual level characteristics are proposed to be adjusted for in the analysis – a description of what will be included and how these factors will be measured is needed
- The proposed analysis using mixed effects models doesn’t align with the sample size calculation – which seems to suppose a pre-post design. Please align your planned analyses and sample size calculations. 

Is the study design appropriate for the research question?

Partly

Is the rationale for, and objectives of, the study clearly described?

Yes

Are sufficient details of the methods provided to allow replication by others?

No

Are the datasets clearly presented in a useable and accessible format?

Not applicable

Reviewer Expertise:

Perinatal epidemiology, epidemiological methods

I confirm that I have read this submission and believe that I have an appropriate level of expertise to confirm that it is of an acceptable scientific standard, however I have significant reservations, as outlined above.

# Reviewer report: Reviewer response for version 3

**DOI:** 10.21956/gatesopenres.17706.r39423

**Published:** 2025-02-25

**Authors:** Christine McCourt

**Affiliations:** 1 City University of London, London, England, UK

I have read the authors’ explanations of the amendments made and I am satisfied on this basis that they have now addressed the recommendations.

Is the study design appropriate for the research question?

Yes

Is the rationale for, and objectives of, the study clearly described?

Yes

Are sufficient details of the methods provided to allow replication by others?

Partly

Are the datasets clearly presented in a useable and accessible format?

Yes

Reviewer Expertise:

maternal and infant health; complex interventions

I confirm that I have read this submission and believe that I have an appropriate level of expertise to confirm that it is of an acceptable scientific standard.

# Reviewer report: Reviewer response for version 3

**DOI:** 10.21956/gatesopenres.17706.r39421

**Published:** 2025-02-14

**Authors:** Yibeltal Kiflie

**Affiliations:** 1 Jimma University(Ringgold ID: 107839), Jimma, Oromia, Ethiopia; 2 MERQ Consultancy PLC, Addis Ababa, Ethiopia

I thank the authors for their efforts in improving the manuscript. They have effectively addressed most of the comments I provided and have resolved my concerns.

 Thank you!

Is the study design appropriate for the research question?

Yes

Is the rationale for, and objectives of, the study clearly described?

Partly

Are sufficient details of the methods provided to allow replication by others?

Yes

Are the datasets clearly presented in a useable and accessible format?

Not applicable

Reviewer Expertise:

Health systems research, program evaluation

I confirm that I have read this submission and believe that I have an appropriate level of expertise to confirm that it is of an acceptable scientific standard.

# Reviewer report: Reviewer response for version 2

**DOI:** 10.21956/gatesopenres.17691.r38686

**Published:** 2025-01-22

**Authors:** Mariame Ouedraogo

**Affiliations:** 1 University of Toronto, Toronto, Canada

I would like to thank the authors for submitting this very interesting study protocol. It focuses on the implementation of group ANC at the health post level in the Amhara Region of Ethiopia. The intervention aims to determine whether group ANC is a feasible, effective, and acceptable way to promote the uptake of ANC services and facility-based delivery. The paper is well-written and presents a strong rationale for this intervention. I appreciate the inclusion of detailed figures and tables, which enhance the understanding of the study design and deployment.

 My comments are relatively minor and aim to enhance the manuscript's clarity, specifically the reporting of the methods.

- The data collection section could be improved by organizing the information under “quantitative data collection” and “qualitative data collection” sub-headings, similar to the format used in the data analysis section. Additionally, providing more details about the main themes of the baseline and endline surveys would be helpful.
- On page 4, the authors state that "up to two additional pregnant women who meet study criteria can be added to a G-ANC cohort if added to the cohort by the second G-ANC meeting." A clear rationale for extending the sample beyond the initial 10 women would be helpful, or it could simply be rephrased to indicate recruiting up to 12 women per health post.
- Also, on page 4, the authors mention that "the first half of clusters will have a choice to continue G-ANC with a second cohort of women during T3." It would be helpful to clarify what this entails and how it will be monitored. With T3, the authors seem to have an opportunity to investigate the maintenance or integration of group ANC4 among the health posts that received the intervention at T3. Some data collection, even if only with healthcare providers, could be valuable to assess whether health posts were able to integrate group ANC into their practice or if they discontinued it after the intervention concluded.
- On page 7, the authors state that the study will be conducted in 12 health centers. For greater clarity, I suggest that the authors specify that the intervention will be delivered at health posts. While I understand that health posts are within the catchment area of health centers, using more precise and consistent language would enhance clarity. In other parts of the document, the authors call health centers "clusters," which may be a more appropriate term.
- On page 10, the authors write that "all data will be de-identified for those who decline study participation." Does this mean the data will be deleted? It would be beneficial to detail how the data from those who refuse to participate will be used and interpreted.
- Also, on page 10, it is written that "11 questions on satisfaction during the last antenatal care will be asked about the antenatal care received during your last pregnancy and the current pregnancy for this G-ANC." This sentence needs some rephrasing. Is the goal to compare satisfaction from the last pregnancy with that of the current pregnancy? If so, what about women who are having their first child? In line with my first comment, I suggest the authors provide more details on their data collection approaches, particularly as they relate to measuring client satisfaction with the intervention.
- Based on the background information provided by the authors regarding the country's specific ANC goals, I expected that the proportion of women attending at least eight visits would be one of the study outcomes. Is the number of ANC contacts collected?
- I agree with the authors that conducting descriptive subgroup analyses will be valuable in seeing if outcomes vary by key socio-demographic characteristics. The authors mentioned considering marital status, education, and economic status. I wondered if they also included age and parity, given that the eligibility criteria include all pregnant women starting at age 15.
- It would also be helpful to include a specific section on the “pause and reflect” component, highlighting specific reporting outcomes during the pause and reflect sessions to ensure fidelity is measured throughout the project. Additionally, it is unclear if and how that information will be used in the analysis to compare implementation between T2 and T3.
- I did not see any information on how women who develop pregnancy complications will be handled. The paper is not very clear on that point. Would they be referred for further care and excluded from the intervention for the remainder of the study? Also, what about women enrolled in the intervention who seek ANC outside of the group sessions? How would that information be collected and included in the analyses?

Thank you once more for an excellent paper on a very important intervention.

Is the study design appropriate for the research question?

Yes

Is the rationale for, and objectives of, the study clearly described?

Yes

Are sufficient details of the methods provided to allow replication by others?

Partly

Are the datasets clearly presented in a useable and accessible format?

Not applicable

Reviewer Expertise:

Epidemiology, maternal and child health, implementation sciences

I confirm that I have read this submission and believe that I have an appropriate level of expertise to confirm that it is of an acceptable scientific standard, however I have significant reservations, as outlined above.

# Reviewer report: Reviewer response for version 2

**DOI:** 10.21956/gatesopenres.17691.r38845

**Published:** 2024-12-26

**Authors:** Meresa Berwo Mengesha

**Affiliations:** 1 Adigrat University, Adigrat, Ethiopia

1. The use of a stepped wedge study design is commendable, as it allows for adaptive management during the intervention.
2. To maintain clarity and consistency throughout the document, please use "Group Antenatal Care models." If you are aware of other models, such as participatory and centering pregnancy models, how do you plan to complement them with your model of care?
3. I am interested in understanding the historical background of Group Antenatal Care (G-ANC) and why this model is primarily implemented in former British colonies in Sub-Saharan Africa. It is encouraging to see the feasibility and effectiveness of this model being tested in low-resource settings like Ethiopia. However, I would also like to know if the study includes maternal and perinatal outcomes associated with this model of care, in addition to the continuum of maternal health care services, such as attendance at antenatal care and utilization of institutional delivery. Furthermore, if the follow-up study is postponed to the postpartum period, it would be beneficial to include the utilization of other services, such as postnatal care and postpartum family planning, as there is a scarcity of data on these topics.
4. One critical aspect of the published works is the blinding and concealment of treatment and outcome assessors. How do you plan to ensure that those delivering treatments remain blind to treatment assignment and that outcome assessors are also blinded? Please clearly state this in your study plan.
5. While it is widely accepted that this model can increase retention in services, how can it also enhance early entry into antenatal care bookings? Can you support this assertion with relevant literature?
6. What interests you in conducting a predetermined subgroup analysis based on marital status, education, and economic status?

Is the study design appropriate for the research question?

Yes

Is the rationale for, and objectives of, the study clearly described?

Yes

Are sufficient details of the methods provided to allow replication by others?

Partly

Are the datasets clearly presented in a useable and accessible format?

Not applicable

Reviewer Expertise:

Maternal and Perinatal Health research, Perinatal mental health and fetal medicine.

I confirm that I have read this submission and believe that I have an appropriate level of expertise to confirm that it is of an acceptable scientific standard.

# Reviewer report: Reviewer response for version 2

**DOI:** 10.21956/gatesopenres.17691.r38690

**Published:** 2024-12-12

**Authors:** Angela M Bengtson

**Affiliations:** 1 Department of Epidemiology, Emory University, Atlanta, Georgia, USA

Thank you for the opportunity to review this important study protocol. I've tried to make comments below to help clarify the design and analysis of the proposed study. 

**Abstract**

- Main outcomes of the study and the length of follow up for participants or the intervention should be included.

**Paper**

- Recommend describing the goal of the study as to “evaluate the acceptability, feasibility, and effectiveness of group ANC (G-ANC) delivered by HEWs at the health post level, compared to usual ANC …” since the intervention being tested here is whether HEW can deliver G-ANC.
- **Methods**
  - **Design**
    - The description of the design needs to be clearer. For example, the abstract indicates that 6 health centers, each with 6 health posts under them, will be randomized in a stepped wedge design to the intervention. However, the methods indicates only 3 (?) health posts per cluster will be included.
      - The design is clustered at the health center level, so there are 6 clusters, but Figure 1 in the Methods lists 12 clusters, which is confusing. I would suggest removing the ‘Sequence’ column as it ‘s not clear what that represents, align the number of clusters with the correct number of health centers, and then making it clear in the text how the data from T1 will be used for the second cluster of health centers randomized to the intervention at T3.
    - What is the rationale for the pre-trial pilot? More specifically, what will happen if the pilot is done and the intervention is *not* found to be feasible or acceptable? A bit more background on the rationale for why this is likely to work may be useful in the introduction. In addition, the rationale for the pilot phase could be framed more around pre-testing intervention materials, etc.
    - I recommend describing the type of care participants will receive based on the time period. For example in T1 (i.e. not prior to the intervention) all women will receive individual ANC. And then in T2, women in the second set of clusters will also continue to receive individual ANC as the control condition?
    - There’s also confusion about the number of health posts included – the abstract suggests 6 per health center, but the Methods suggests 3 of 5 will be selected and the sample size section indicates 3 health posts per health center. Please clarify and align.
      - What the inclusion/exclusion for health posts will be and how they will be selected also needs to be included
      - More specific inclusion/exclusion criteria for the participants is also needed – are there restrictions based on age for pregnant women or complications (GPNC is intended for healthy, normal pregnancies, not those considered high risk), or plans to attend ANC?
    - The qualitative interviews need to be described in the design of the study, along with the design of the pilot/trial
  - **Intervention**
    - What is the rationale for the following “Up to two additional pregnant women who meet study criteria can be added to a G-ANC cohort if added to the cohort by the second G-ANC meeting”?
    - How will women who develop complications, and may be referred to the health center level, be accounted for or handled?
    - If G-ANC is going to be delivered at the “health posts that offer integrated point-of-care testing (POCT) to screen for anemia, syphilis, and asymptomatic bacteriuria (ASB)” then this needs to be part of the inclusion criteria for selecting eligible health posts.
      - NB: Table 1 lists POCT as an inclusion criteria for health centers but not health posts, which doesn’t align with the text
      - Exclusion criteria for pregnant women around conditions that may complicate study interpretation or objectives needs to be more specific as to what would meet this criteria (high blood pressure, gestational diabetes, obesity, mental health issues?)
    - Ahh, I see some information on inclusion/exclusion is in Table 1. This needs to be aligned with the text to either more clearly reference this table or to clarify the inclusion/exclusion criteria being used
      - A point of clarification – health centers have inclusion criteria because they are the unit of randomization, but all participants will be enrolled at the health post level (i.e. no one will be enrolled at the health center) – is that correct?
  - **Training**
    - Please describe who will conduct the training for HEWs and midwives
    - Please also provide more detail on how the midwives from health centers will provide mentorship to the HEWs – what will this include and at what level will the midwives be involved?
  - **Statistical analysis**
    - Please be explicit about which time periods will be compared when estimating primary outcomes – for example is T1 included for health centers not randomized to the intervention until T3? Are comparisons only being made within a given time period or across time periods for intervention and control conditions?
      - For example – are the proportions in Table 4 suggest a pre-post design; is the expectation that health centers who receive the intervention in T2 would be compared to their T1, rather than intervention and control compared at T2? Please clarify
    - Outcome definitions need to be more specific – i.e. The proportion of women with at least 4 ANC visits by time period? By intervention vs control? And how will satisfaction with ANC be measured?
    - In addition, some discussion around how the pause and reflect sessions will be used to adjust intervention content and if and how that will be handled at the analysis phase is needed. If comparisons are only planned by intervention and control groups within a given time period (which needs clarification) then this may not be an issue. But if there is interest in analyzing all intervention period together and there are major changes to the intervention during the pause and reflect period, this would not be appropriate.
    - It’s not clear in the design that randomization is stratified by location and client volume – please clarify this in the design and discuss in the statistical approach if and how this will be taken into account.
  - The study timeline of 18 months stated in the text doesn’t align with Figure 2, which lists a 3-4 month pilot, pause and reflect period and 6 months for each of the T1-T3 time periods
  - More information on the types of data collected at the baseline and endline survey is needed – is only sociodemographic information included?
    - NB: facility and individual level characteristics are proposed to be adjusted for in the analysis – a description of what will be included and how these factors will be measured is needed
  - Some of the information in the statistical analysis and quantitative data analysis sections are repetitive and could be streamlined into one section
    - The proposed analysis using mixed effects models doesn’t align with the sample size calculation – which seems to suppose a pre-post design
    - As mentioned above, if data will be analyzed across intervention time periods, how will intervention adaptations or changes resulting from the pause and reflect periods be considered?

Is the study design appropriate for the research question?

Partly

Is the rationale for, and objectives of, the study clearly described?

Yes

Are sufficient details of the methods provided to allow replication by others?

No

Are the datasets clearly presented in a useable and accessible format?

Not applicable

Reviewer Expertise:

Perinatal epidemiology, epidemiological methods

I confirm that I have read this submission and believe that I have an appropriate level of expertise to confirm that it is of an acceptable scientific standard, however I have significant reservations, as outlined above.

# Reviewer report: Reviewer response for version 2

**DOI:** 10.21956/gatesopenres.17691.r38345

**Published:** 2024-11-12

**Authors:** Yibeltal Kiflie

**Affiliations:** 1 Jimma University(Ringgold ID: 107839), Jimma, Oromia, Ethiopia; 2 MERQ Consultancy PLC, Addis Ababa, Ethiopia

Dear editor, I forwarded the comments and suggestions below during my initial review. The revised version does not address these comments, and the authors did not respond to any of them.

**Title**

 Evaluation of the feasibility, acceptability, and impact of Group Antenatal Care at the health post level on continuation in antenatal care and facility-based delivery in Ethiopia using a cluster randomized stepped-wedge design: Study protocol

**Comments and Suggestions**

**General Comments**

- The study intends to explore a potential solution for one of the major challenges in the provision of maternal health care in Ethiopia. The protocol also provides comprehensive information regarding key aspects of a trail.
- The study was declared “Terminated” at clinicaltrials.gov because of the current security challenges in Amhara region. How do the authors view the importance of publishing the current protocol here? The authors also need to be more transparent in reporting trial registration.
- Quantitative baseline and endline surveys are already conducted for T1 and T2. Data collection for T3 is what is pending. What is the purpose of publishing a protocol paper at this stage? Given the prolonged security challenges in the region, as an alternative, the authors may consider publishing their findings from analysis of the data from T1 and T2 baseline and endline surveys with a detailed description of the original protocol in the methods section.
- Having completed data collection for T1 and T2 and a plan to pause and reflect, what changes were made to the original protocol based on learnings from the piloting, T1, and T2 data collection activities?

**Introduction**

- The authors need to review data and research on ANC client flow/case load at the health post level and analyze what that means regarding the feasibility of G-ANC at the health post level. Will health posts in Ethiopia have enough number of ANC clients to implement G-ANC?
- The possible causes of losses to the maternal health care continuum of care in Ethiopia and the plausibility of the hypothesis that G-ANC will improve retention is not adequately presented in the introduction section.
- Some of the contents presented in the introduction section need a government document as a reference. This includes contents like what maternal health services are provided at HC and at HP, staffing patterns of health posts.

**Study Design**

- The paper provided a clear description of the stepped wedge cluster randomized controlled trial. However, they also added a section that describes piloting in 5 health posts to determine feasibility. As the authors have already determined feasibility, I suggest taking all the information about feasibility (including how feasibility was assessed and their findings) to the introduction section.

**Study Setting**

- If the authors are intending to continue the study even with the current security challenges, the security situation should be clearly described here.

**Inclusion and exclusion criteria**

- Currently, health posts in Ethiopia are classified in to two categories – basic and comprehensive. Even though the health posts are not yet fully restructured into these two new categories, it is important that the authors mention which categories of health posts are included in their study.

**Intervention**

- How similar/different are contents of the G-ANC meetings with that of standard ANC sessions? Table 2 will be more informative if it is revised to include this information instead of presenting just the contents of the G-ANC sessions.

**Recruitment**

- One of the four recruitment strategies is “3) All new ANC attendees in the study HC”. Is this a typo or do the authors consider health centers as recruitment sites for their study? If recruitment is happening at health centers, will clients be referred to health posts just for the purpose of the study?

Is the study design appropriate for the research question?

Yes

Is the rationale for, and objectives of, the study clearly described?

Partly

Are sufficient details of the methods provided to allow replication by others?

Yes

Are the datasets clearly presented in a useable and accessible format?

Not applicable

Reviewer Expertise:

Health systems research, program evaluation

I confirm that I have read this submission and believe that I have an appropriate level of expertise to state that I do not consider it to be of an acceptable scientific standard, for reasons outlined above.

# Reviewer report: Reviewer response for version 2

**DOI:** 10.21956/gatesopenres.17691.r38261

**Published:** 2024-10-23

**Authors:** Christine McCourt

**Affiliations:** 1 City University of London, London, England, UK

Thanks for providing an overview of your amendments to the protocol. These mostly address my previous review feedback but I would recommend some additional minor amendments as follows:

 Abstract – suggest you change ‘facility-based delivery’ to ‘birth with a skilled birth attendant’ as this has a better fit with the evidence. The text can explain that in the Ethiopian context this implies facility-based birth but for consistency of language in the abstract you could say something like: ‘In Ethiopia skilled birth attendance is only available in health facilities’, therefore….

 (this then helps to make clear why you have focused on facility births)

 You have clarified the role and scope of health centres and health posts and who works in each but it would also be helpful to clarify whether health posts provide any birth care or just antenatal (I assume just anc but it is not explicit). Also, it remains unclear whether the comparison/control group care is also delivered in the same setting by the same people (HEWs) with the only difference being that it is individual.

 Under randomisation – please spell out the acronym POCT as I don’t think was used before.

 A few additional points to consider re data:

 Qualitative data – you have specified the software to be used but not the approach – will this be thematic? Also an you outline who will be included in qualitative data collection and the specific methods you plan to use?

 Questionnaires – for assessing empowerment you might want to consider using the PRES scale (developed by Klima et al., 2015) [Ref 1] as this is being used in some other group care studies and this would facilitate comparison of experiences across different settings and studies.

Is the study design appropriate for the research question?

Yes

Is the rationale for, and objectives of, the study clearly described?

Yes

Are sufficient details of the methods provided to allow replication by others?

Partly

Are the datasets clearly presented in a useable and accessible format?

Yes

Reviewer Expertise:

maternal and infant health; complex interventions

I confirm that I have read this submission and believe that I have an appropriate level of expertise to confirm that it is of an acceptable scientific standard, however I have significant reservations, as outlined above.

## References

[1] : Development of the Pregnancy-related Empowerment Scale. *Nursing and Health* .2015;3(5) : 10.13189/nh.2015.030503 120-127 10.13189/nh.2015.030503

# Reviewer report: Reviewer response for version 1

**DOI:** 10.21956/gatesopenres.16534.r36448

**Published:** 2024-07-24

**Authors:** Yibeltal Kiflie

**Affiliations:** 1 Jimma University(Ringgold ID: 107839), Jimma, Oromia, Ethiopia; 2 MERQ Consultancy PLC, Addis Ababa, Ethiopia

**General Comments**

- The study intends to explore a potential solution for one of the major challenges in the provision of maternal health care in Ethiopia. The protocol also provides comprehensive information regarding key aspects of a trail.
- The study was declared “Terminated” at clinicaltrials.gov because of the current security challenges in Amhara region. How do the authors view the importance of publishing the current protocol here? The authors also need to be more transparent in reporting trial registration.
- Quantitative baseline and endline surveys are already conducted for T1 and T2. Data collection for T3 is what is pending. The purpose of publishing a protocol paper at this stage is not clear. Given the prolonged security challenges in the region, as an alternative, the authors may consider publishing their findings from analysis of the data from T1 and T2 baseline and endline surveys with a detailed description of the original protocol in the methods section. 
- Having completed data collection for T1 and T2 and a plan to pause and reflect, what changes were made to the original protocol based on learnings from the piloting, T1, and T2 data collection activities?

**Introduction**

- The authors need to review data and research on ANC client flow/case load at the health post level and analyze what that means regarding the feasibility of G-ANC at the health post level. Will health posts in Ethiopia have enough number of ANC clients to implement G-ANC?
- The possible causes of losses to the maternal health care continuum of care in Ethiopia and the plausibility of the hypothesis that G-ANC will improve retention is not adequately presented in the introduction section.
- Some of the contents presented in the introduction section need a government document as a reference. This includes contents like what maternal health services are provided at HC and at HP, staffing patterns of health posts.

**Study Design**

- The paper provided a clear description of the stepped wedge cluster randomized controlled trial. However, they also added a section that describes piloting in 5 health posts to determine feasibility. As the authors have already determined feasibility, I suggest taking all the information about feasibility (including how feasibility was assessed and their findings) to the introduction section.

**Study Setting**

- If the authors are intending to continue the study even with the current security challenges, the security situation should be clearly described here.

**Inclusion and exclusion criteria**

- Currently, health posts in Ethiopia are classified in to two categories – basic and comprehensive. Even though the health posts are not yet fully restructured into these two new categories, it is important that the authors mention which categories of health posts are included in their study.

**Intervention**

- How similar/different are contents of the G-ANC meetings with that of standard ANC sessions? Table 2 will be more informative if it is revised to include this information instead of presenting just the contents of the G-ANC sessions.

**Recruitment**

- One of the four recruitment strategies is “3) All new ANC attendees in the study HC”. Is this a typo or do the authors consider health centers as recruitment sites for their study? If recruitment is happening at health centers, will clients be referred to health posts just for the purpose of the study?

Is the study design appropriate for the research question?

Yes

Is the rationale for, and objectives of, the study clearly described?

Partly

Are sufficient details of the methods provided to allow replication by others?

Yes

Are the datasets clearly presented in a useable and accessible format?

Not applicable

Reviewer Expertise:

Health systems research, program evaluation

I confirm that I have read this submission and believe that I have an appropriate level of expertise to state that I do not consider it to be of an acceptable scientific standard, for reasons outlined above.

# Reviewer report: Reviewer response for version 1

**DOI:** 10.21956/gatesopenres.16534.r36340

**Published:** 2024-07-17

**Authors:** Christine McCourt

**Affiliations:** 1 City University of London, London, England, UK

Abstract

 A key focus is stated as level of facility-based delivery. This is a common target, but it isn’t a direct indicator of outcomes and may be mediated by birth attendant – in many LMIC settings, and indeed high-income countries, giving birth outside a ‘facility’ means without skilled birth attendance. It is entirely plausible that the key issue is skilled birth attendance. We know that birth outwith hospitals in high income settings with integrated care systems and attendance by a qualified midwife is very safe. The safety implications may of course vary by geographical (e.g. difficulty of transfer to hospital in the event of complications/lack of transport) as well as health system context (qualified midwives available to attend birth at home and fully integrated in the care system, risk assessment, referral systems for those with risk factors), but this is something to bear in mind in your study.

 Introduction

 There is a good brief explanation of health extension workers but no explanation of what is meant by health professionals – stated as HPs – it would be helpful to explain which cadre of HP staff health centres. The relationship between the HEWs and HPs would also benefit from more clarification – currently it just says 2 assigned to 1- can you explain a little more how this works? Who are they assigned to – a midwife? Do the HPs visit the health posts or are only based at health centres?

 The intervention is explained well given the conciseness and the testing of the model as facilitated in health posts by HEWs was clear but the control care was not made clear – will control group women receive individual visits at health posts with HEWs? Or will this be different in some other way? (Table 1 does help with this but I still think it could be made more explicit in the text what the control group care is.)

 In methods, likewise, the schedule of care in the intervention group is explained well but there is a lack of information about control/standard care – do they receive the same number and intervals of anc visits? How long are the individual visits? Is the testing kit being used in both intervention and control groups?

 The training for the HEWs is explained briefly and the midwives who will mentor them will be given mentorship training, but will these midwives also have had the group-care facilitation training or any practice with these so they understand the way the approach works?

 The guide for visits seems heavily focused on understanding risks, which is obviously important, but will the visit plans not include health promotional content such as healthy eating and self-care? (Actually table 3 suggests it will be this isn’t clear from the text.

 Methods

 It would be helpful to provide a brief overview of what the pilot study will involve – this is mentioned (and is a good idea to do) but with no information about what it will involve, for how long. Etc. There is some information further down, but this gives the numbers but no detail about methods involved – for example will HEWs and midwives be interviewed to understand their experience, women involved? Any observation of pilot groups?

 The Step Wedge Cluster trial design is appropriate and described well.

 Outcome measures – as mentioned above use of facility-based birth as a primary outcome is understandable given it is a key national / international target but it is a proxy outcome. You haven’t included any direct clinical outcomes in your analysis plan – what about gestational age or birthweight or a composite healthy baby and/or healthy mother outcome?

 How will empowerment, self-efficacy and satisfaction be assessed? Are you planning to use validated or established tools for these during the interviews with women? If so, which ones?

 If not, how will you approach this?

Is the study design appropriate for the research question?

Yes

Is the rationale for, and objectives of, the study clearly described?

Yes

Are sufficient details of the methods provided to allow replication by others?

Partly

Are the datasets clearly presented in a useable and accessible format?

Yes

Reviewer Expertise:

maternal and infant health; complex interventions

I confirm that I have read this submission and believe that I have an appropriate level of expertise to confirm that it is of an acceptable scientific standard, however I have significant reservations, as outlined above.
